# N-acetyl cysteine ameliorates aortic fibrosis by promoting M2 macrophage polarization in aging mice

**DOI:** 10.1080/13510002.2021.1976568

**Published:** 2021-09-17

**Authors:** Qing-yi Zhu, Shi Tai, Liang Tang, Yi-chao Xiao, Jian-jun Tang, Ya-qin Chen, Li Shen, Jia He, Ming-qi Ouyang, Sheng-hua Zhou

**Affiliations:** Department of Cardiovascular Medicine, The Second Xiangya Hospital, Central South University, Changsha, People’s Republic of China

**Keywords:** Vascular fibrosis, oxidative stress, macrophage polarization, vascular aging, NAC, inflammaging, M1, M2

## Abstract

**Background:** Vascular fibrosis is a universal phenomenon associated with aging, and oxidative stress plays an important role in the genesis of vascular damage in line with the aging process. However, whether antioxidants can ameliorate vascular fibrosis remains unclear.

**Objectives:** The present study was to determine antioxidant N-acetylcysteine (NAC) could ameliorates aortic fibrosis in aging wild-type C57BL/6 mice.

**Methods**: The aortas were harvested from both 12-week and 60-week wild-type mice. The 60-week mice were treated with and without the NAC for 12 weeks starting at the age of 48 weeks. Hematoxylin and eosin (H&E) staining and Masson's trichrome staining of aortic samples were performed, and the levels of reactive oxygen species (ROS), RNA expression of GAPDH, TNF-α, MCP-1, IL-6, IL-10, IL-4, SIRT-1, SIRT-3, FOXO-1, and macrophage polarization were determined.

**Results:** There is a positive relationship between collagen deposition and the M1/M2 macrophage ratio in the aortic wall of aged wild-type C57BL/6 mice. The higher collagen area percentage in the aortas of 60-week-old mice than in 12-week-old mice was reversed by NAC. NAC could not impact the total number of macrophages, but partly promoted M2 macrophage polarization. By performing qRT-PCR using aortic samples from these mice, we identified that SIRT-1, SIRT-3, FOXO-1 could be somehow responsible for aging-related fibrosis.

**Conclusions:** NAC ameliorates aortic fibrosis in aging wild type mice partly by promoting M2 macrophage polarization.

## Introduction

Populations around the world are rapidly aging. Although an increase in the elderly population indicates improvements in global public health, this population may experience a diminished quality of life because of the negative impacts of aging-related diseases [1]. Vascular aging is a universal process associated with aging-related diseases including cardiovascular disease, stroke, and cancer [2,3]. Physiological aging also shows progressive vascular remodeling and involves changes in collagen deposition [4] that leads to fibrosis. Arterial fibrosis is associated with the development of many age-associated disease like hypertension, atherosclerosis, diabetes, dementia, and renal fibrosis [3,5]. Among these findings, clinical studies have identified age as one of the most potent determinants of changes in arterial structure [6]. A cross-sectional study identified that both vascular fibrosis and some degree of oxidative stress developed in the elderly [7].

A considerable number of published evidence supports the involvement of oxidative stress in the genesis of vascular damage within the aging process [7–11]. The antioxidant N-acetyl cysteine (NAC) has been widely used as a treatment for idiopathic pulmonary fibrosis [12]. It has also been proved to inhibit the development of fibrosis of cardiac [11], kidney [13], liver [14] and ovary [15]. However, whether NAC also has a positive effect on physiological vascular aging and the underlying potential mechanisms are still unknown.

In addition to excessive oxidative stress [16], chronic inflammation is a common cause of vascular dysfunction in aging, also known as ‘inflammaging’ [17]. Macrophages are one of the most abundantly produced immune cells that are involved in the development of pathological changes during inflammation, and play an essential role in several aging-related diseases [18–20]. Macrophages identified in human atheroma are classified as a classical proinflammatory phenotype (M1) or an alternative anti-inflammatory phenotype (M2) [21]. In the present study, we aimed to evaluate whether vascular fibrosis is developed during the physiological aging process in C57BL/6 mice and whether macrophage polarization is involved in improving the antioxidant role of NAC in aging mice.

## Materials and methods

Materials and Methods are available in the *online-only Data Supplement*.

## Results

### NAC treatment could not change the body weight of old mice

The body weight of C57BL/6 mice was significantly higher in the 60-week group than in the 12-week group (12 W: 21.20 ± 1.11 g *vs.* 60 W: 29.48 ± 1.84 g, *p* < 0.001). There was no significant difference between the body weight of mice of the same age with or without 12-week NAC treatment (60 W + NAC: 29.31 ± 2.01 g *vs.* 60 W: 29.48 ± 1.84 g, *p = *0.997).

### NAC treatment alleviates arterial fibrosis associated with aging

**Figure 1.**
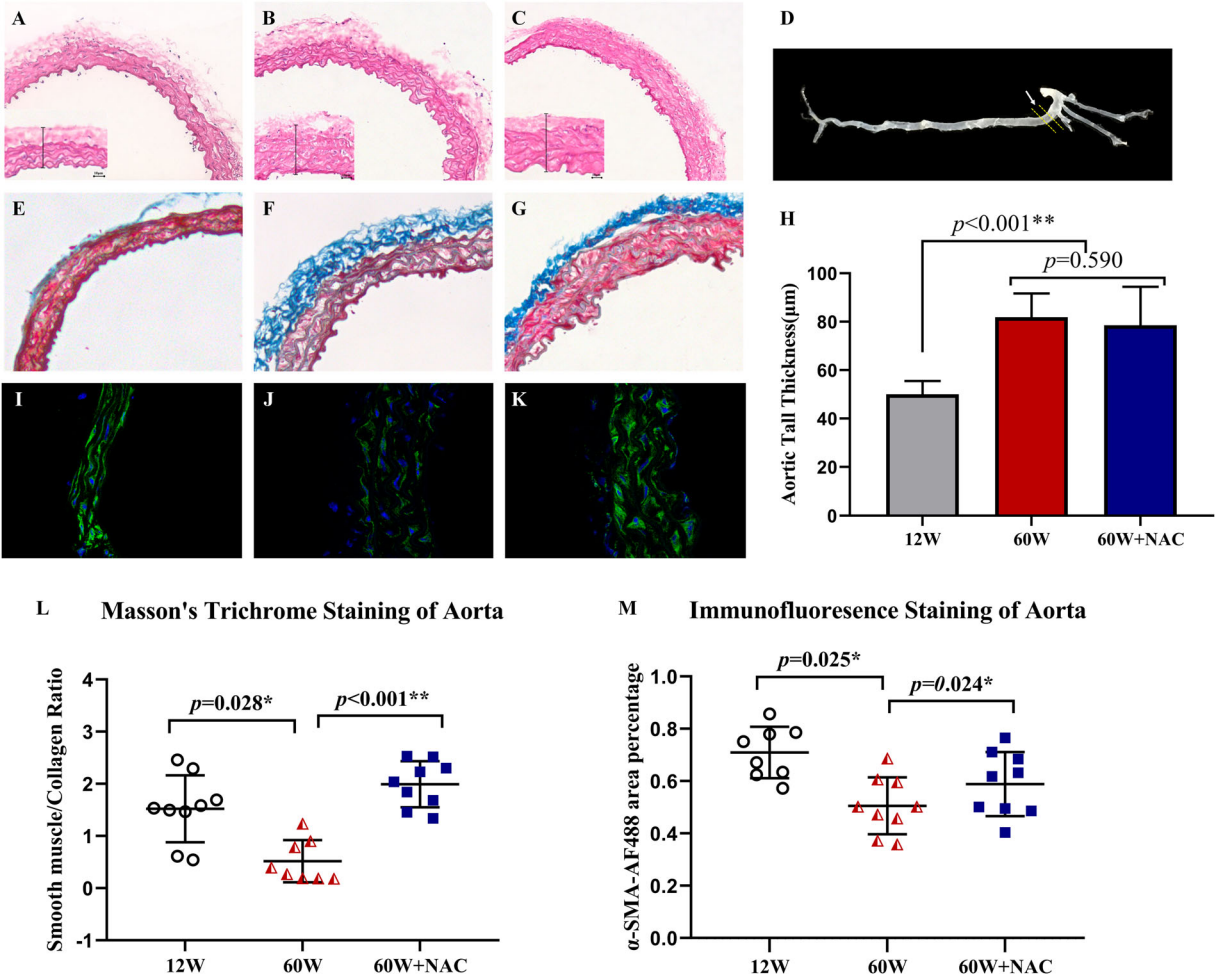
Arterial structure shown by H&E staining (A, B, C) and the extent of fibrosis determined by Masson’s trichrome staining (E, F, G) and α-SMA- AF488 immunofluorescence staining (I, J, K). Figure 1(D) shows the location where the mouse aorta was cut to obtain aortic rings (white arrow). Digital analysis of the wall thickness was compared in Figure 1(H). Digital analysis of the ratio of non-fibrotic areas mainly including smooth muscle (red) and fibrotic areas mainly including collagen (blue) was presented in Figure 1(L). Original magnification was ×10 in figure A–C and E–G and was ×63 in figure I–K. Data were as means ± SD (*n* = 8–9 each). *p < 0.05, **p < 0.01.

Changes in arterial structure and fibrosis were detected by H&E staining, Masson’s trichrome staining and a-SMA-AF488 immunofluorescence staining (Figure 1). The location where the aorta was cut to obtain the aortic ring sections is shown in Figure 1(D). H&E staining showed that the arterial wall was thicker in the older groups (60 W: 81.84 ± 9.88 μm and 60 W + NAC: 78.55 ± 15.89 μm) than in the younger group (12 W: 50.04 ± 5.54 μm) (Figure 1(A–C,H)). The blue color of Masson’s trichrome staining indicates the presence of collagen (Figure 1(E–G)). The ratio of smooth muscle (red) and collagen (blue) (Figure 1(L)) was significantly lower in the older group than in the younger group (12 W: 1.35 ± 0.38 vs. 60 W: 0.52 ± 0.40, *p* = 0.028), and 12-week NAC treatment reversed this aging-related fibrosis: The ratio in the 60 W + NAC group was up to 2.01 ± 0.38 compared to the 60 W group (*p* < 0.001), but there was no difference between 12 and 60 W + NAC groups (*p = *0.081). This phenomenon has also been validated by a-SMA staining (Figure 1(I–K)). The a-SMA- AF488 positive area percentage of aorta slides was smaller in 60 W than 12 W group (*p* = 0.025) and NAC could increase the expression of α-SMA (*p* = 0.024) (Figure 1(M)).

### NAC treatment inhibits ROS production in arterial walls

**Figure 2.**
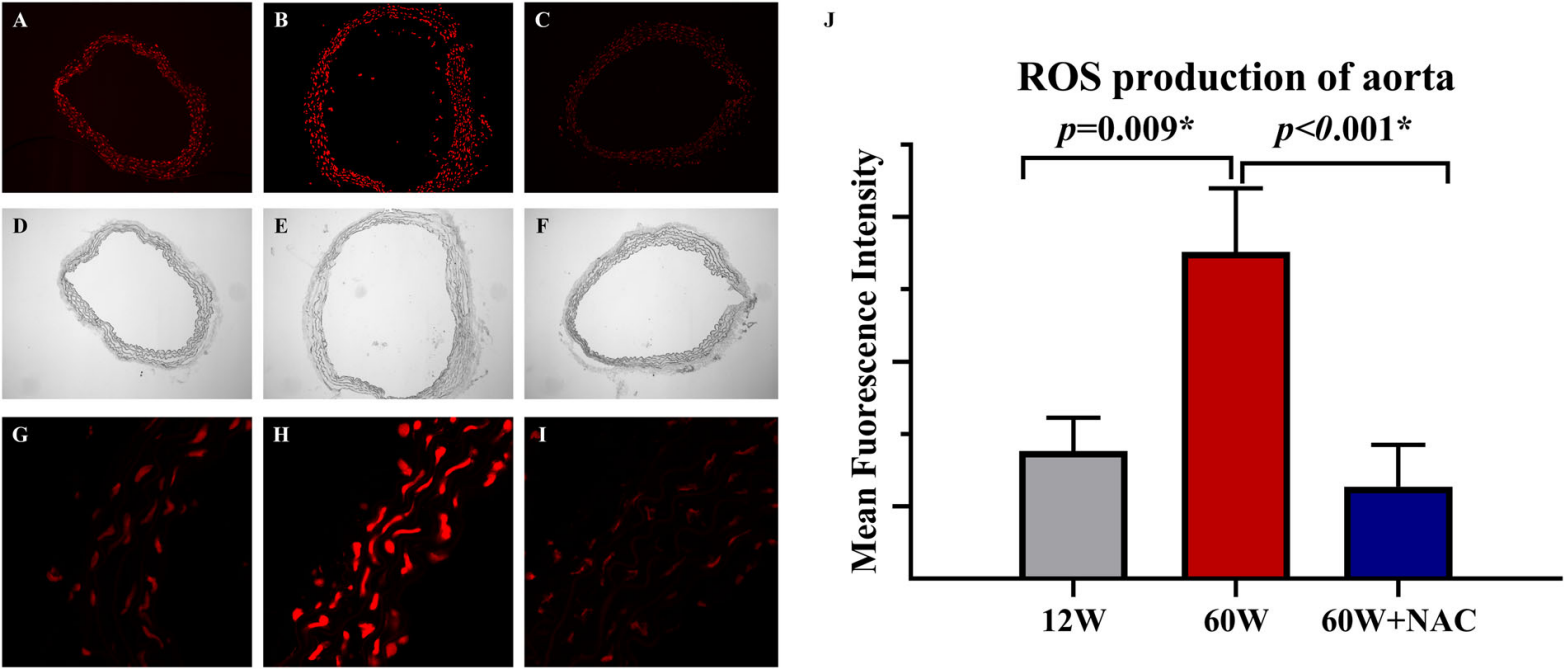
Representative dihydroethidium staining (A–C) and quantitative analysis of the red signal (J) in aortas from different age groups and after NAC treatment. The aortic ring in blank field was shown in figure D-F. Original magnification was ×10 in figure A–F and was ×63 in figure G–I. Data were as means ± SD (*n* = 8 each). *p < 0.05, **p < 0.01.

Accumulation of reactive oxygen species (ROS) in the aorta was detected by dihydroethidium (DHE) staining (Figure 2(A–C,G–I)). A significant increase in ROS production was found in the aorta of older mice and 12-week NAC treatment completely inhibited ROS production in the aorta of these mice (Figure 2(J)).

### Effect of aging and NAC treatment on macrophage polarization in the aortic wall

**Figure 3.**
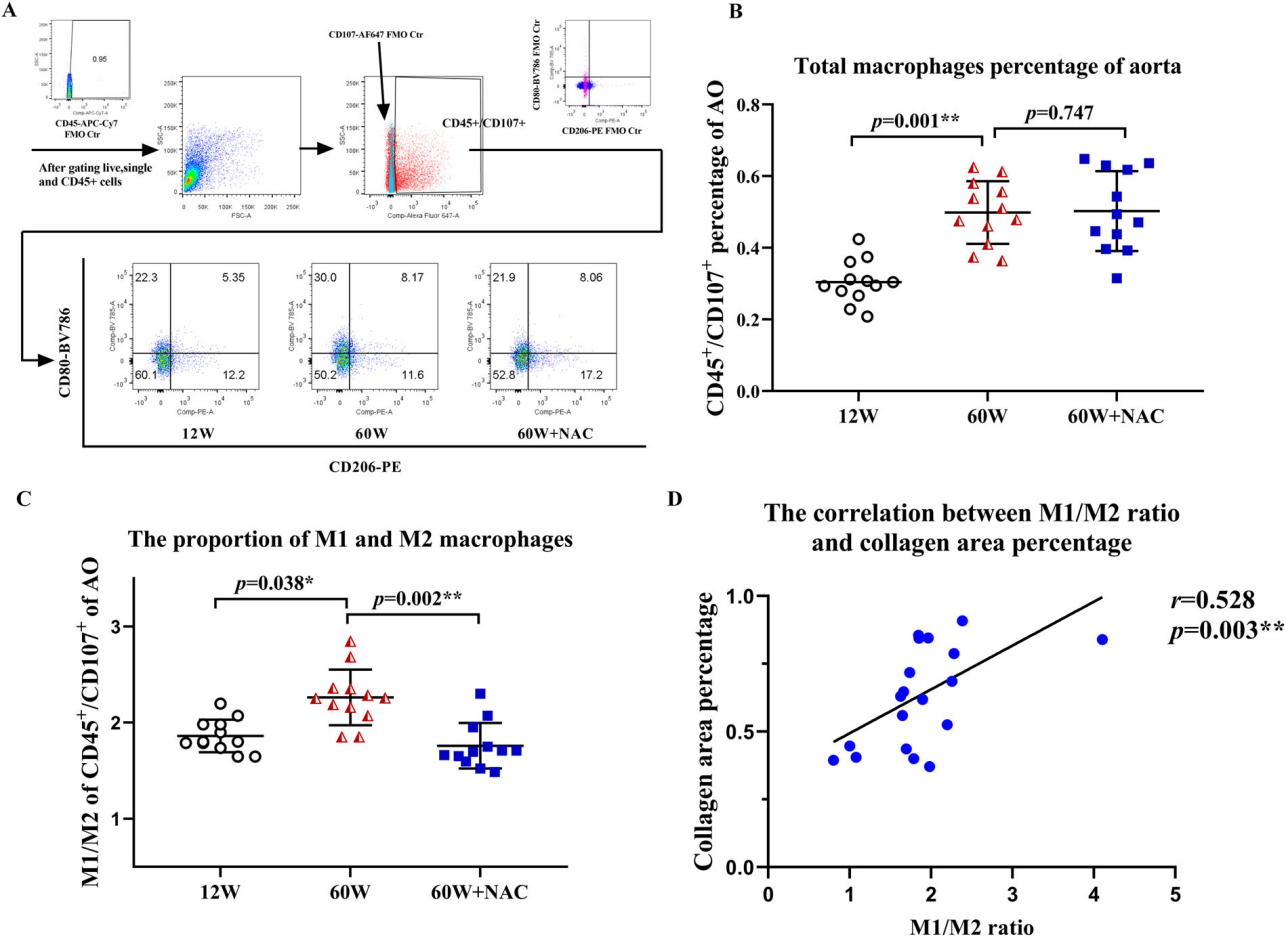
Digested cells of the whole aortic tissue were analyzed by flow cytometry. Gates set up were based on FMO controls (A). Live cells, CD45 positive leukocytes, CD45 and CD107 double positive macrophages were obtained. M1 and M2 populations were identified by staining anti-CD80 BV786, and anti-CD206 PE antibodies further (A). Figure 3(B) shows the comparison results of the total number of macrophages between different groups. The total percentages of CD45^+^/CD107^+^macrophages were significantly higher in aging groups (*p* < 0.01). The ratio of M1 (CD45^+^/CD107^+^/CD80^+^/CD206^-^) to M2 (CD45^+^/CD107^+^/CD80^-^/CD206^+^) macrophages (C) was significantly higher in the 60 W group than in the 12 W group (*p = *0.038), and the 12-week NAC treatment reversed this phenomenon (*p = *0.002). A positive relationship (D) between the M1/M2 macrophage ratio and collagen areas percentage stained by Masson’s trichrome in the aortic walls was observed (*r *= 0.528, *p = *0.003). Data were as means ± SD (*n* = 12 each). *p < 0.05, **p < 0.01.

**Figure 4.**
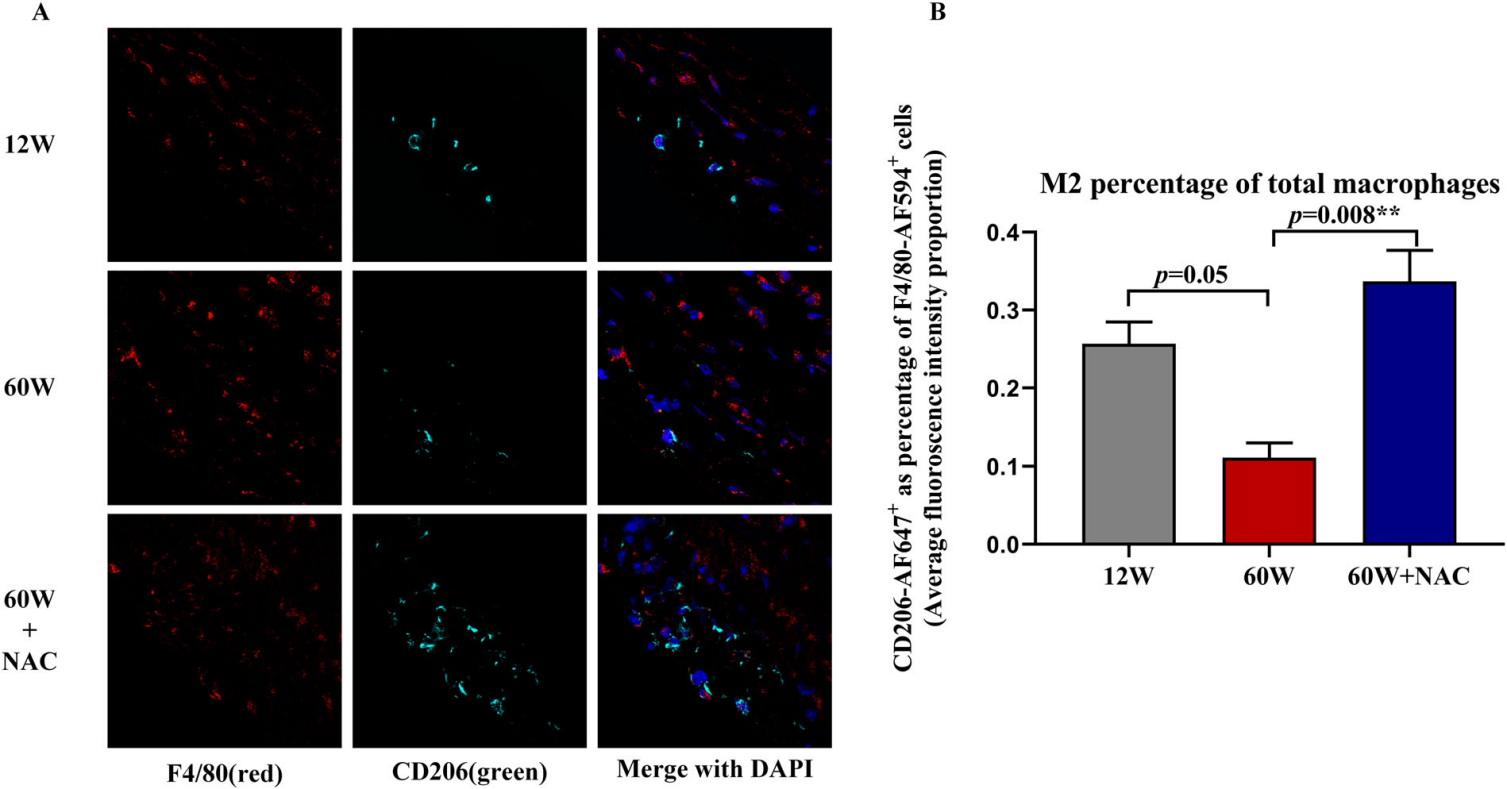
(A) M2 macrophages in the aortic sinus sections were identified by anti-F4/80 AF594 and anti-CD206 AF647 antibodies. F4/80-AF594(red) represented the total macrophages and CD206-AF647(green) represented M2 macrophages. (B) Bar charts show quantification results for the ratio of M2/total macrophages (CD206-AF647^+^ cells/ F4/80-AF594^+^cells), M2 (F4/80^+^/CD206^+^). The ratio in the aorta sinus was lower in 60W group compared to 12W group (*p* = 0.05), 12-week NAC treatment significantly increased this ratio in the aorta sinus (*p* = 0.008) of the 60 W group. Data were as means ± SD (*n* = 8 each). *p < 0.05, **p < 0.01.

The polarization of macrophages in the aortas were analyzed by flow cytometry. 1.0 × 10^6^ cells per aorta were sorted. After staining using a viability dye and treating the cells with anti-CD45 APC-Cy7, single and CD45-positive cells were gated as leukocytes (Figure 3(A)). CD45^+^ and CD107^+^ double positive cells were identified as macrophages in this study. The percentage of CD45^+^/CD107^+^ macrophages of all aortic cells in group12 W, 60 W and 60 W + NAC is 0.30 ± 0.06%, 0.51 ± 0.13% and 0.49 ± 0.13% separately. The total percentage of CD45^+^/CD107^+^ was significantly higher in aging groups both treated with NAC (*p *= 0.002) or without NAC (*p *= 0.001) compared to 12 W group. But no difference was noted between the two 60 W groups (Figure 3(B)) showing total macrophage numbers increased with aging and not affected by NAC. A positive relationship was noticed between the ratio of M1 (CD45^+^/CD107^+^/CD80^+^/CD206^-^) to M2 (CD45^+^/CD107^+^/CD80^-^/CD206^+^) macrophages and the collagen area percentage of Masson’s trichrome staining in the aortic wall (*r *= 0.528, *p *=* *0.003, Figure 3(D)). The M1/M2 ratio in both 12 and 60 W + NAC groups were significantly lower than in the 60 W group (Figure 3(C)). Notably, the M2 population marked as CD80^-^/CD206^+^ percentage of CD45^+^/CD107^+^ cells showed a higher trend in the NAC treatment group (60 W + NAC:13.87 ± 1.73% *vs.*60 W: 11.99 ± 1.35%, *p *=* *0.035). This observation was further confirmed by immunostaining the M2 population (F4/80-AF594^+^/CD206-AF647^+^ double positive cells) in the aortic sinus sections. NAC treatment led to significantly higher fluorescence intensity of M2 macrophages percentage of total macrophages (F4/80-AF594^+^ cells) (*p* = 0.008) (Figure 4(A,B)), suggesting that NAC may have stimulated M2 polarization during arterial fibrosis associated with aging.

### NAC increase SIRT1, SIRT3, FOXO1 mRNA levels in the aorta of aging mice

The expression of regional inflammatory cytokines TNF-α, IL-6, MCP-1, and IL-4, and anti-inflammatory cytokine IL-10 in the aortic walls was analyzed by qRT-PCR. Only the mRNA expression of MCP-1 was higher in the older group than in the younger group (60 W: 4.83 ± 1.25 *vs.* 12 W: 2.86 ± 1.93, *p = *0.044). The mRNA levels of sirtuin 1 (SIRT1) (60 W: 1.07 ± 0.32 *vs*. 12 W: 1.42 ± 0.42, *p = *0.032) and foxhead box protein O1 (FOXO1) (60 W: 0.79 ± 0.28 *vs.* 12 W: 1.81 ± 0.18, *p = *0.001) decreased in the 60 W group than in the 12 W group but increased in the NAC treatment group (SIRT1: 60 W + NAC: 1.75 ± 0.49, *p* < 0.001, FOXO1: 60 W + NAC: 1.57 ± 0.19, *p = *0.009). The 12-week NAC treatment also increased the sirtuin 3 (SIRT3) gene expression (60 W + NAC: 1.14 ± 0.08 *vs.* 60 W: 0.84 ± 0.20, *p = *0.035) in the aortas of the aging group.

## Discussion

The vascular structure including the intimal-to-media thickness of the artery is strongly associated with aging and is also a predictor of future cardiovascular events [22,23]. Vascular fibrosis involves changes in collagen deposition [7] and a higher percentage of collagen was found in the aortas of the older group in our study (Figure 1). In a previously performed clinical trial, age has been reported to be associated with fibrosis in some, but not all, arterial segments [3]. However, in our study, we only analyzed a part of the aortic arch obtained from mice, which is a limitation of this study. Additionally, the clinical trial showed that age was not significantly associated with fibrosis according to the multivariable analyses performed separately based on muscular and elastic artery data [3]. This observation partially differs from that of our study because of the difference in the types of study models (physiological process of mice observed in our study versus the use of human samples obtained from patients with hypertension or diabetes in the clinical trial). Hence, we suggest that the correlation between age and vascular fibrosis in the arteries of different diameters and with different functions need to be further studied.

The mechanism of aging that causes fibrosis remains unclear but at a molecular level, aging has a strong relationship with oxidative stress damage [23,24]. Oxidative stress impairs the mobility and function of endothelial progenitor cells and enhances cellular senescence [25]. The accumulation of ROS is higher in the aorta of the elderly and can be inhibited by NAC (Figure 2); ROS accumulation may be caused by dysfunctional mitochondria that leads to DNA damage and increased amounts of oxidized proteins [26]. We chose NAC for its exact clinical effect against pulmonary fibrosis, and its effect on aortic fibrosis associated with aging was also confirmed in our study (Figure 1). Other antioxidants may also provide similar benefits in reversing vascular fibrosis [27]; however, we only determined the effect of NAC in our study, which is another limitation. But we believe NAC is unique in all the antioxidants because it has already been proved to decrease fibrosis in other organs like heart [11], lung [12], kidney [13], liver [14] and ovary [15].

We further investigated how NAC improves vascular fibrosis. In addition to oxidative events, chronic inflammation is associated with aging [17] and with arterial stiffness in humans [28]. Macrophages provide the required immune support and play a pivotal role in fibrosis during the healing process after an injury [29]. In the flow cytometry, to identify all leukocytes in the aortas, leukocyte common antigen (CD45) was used [30]. Macrophages can be identified by specific expression of a number of proteins including CD14, CD11b, F4/80 and MAC-1/MAC-3(CD107) [31]. In order to better do the compensation, we choose CD107-AF647 to present the whole macrophages in the flow cytometry. While F4/80 is a widely-used surface marker of macrophage [32], we kept it in the immunofluorescence staining to present all the macrophages and got the same result. CD206, known as mannose receptor, often expressed on M2 activated by interleukin-4 (IL-4) or IL-13[33], is used in both flow cytometry and immunostaining. CD80 is used as the M1 macrophages surface marker as referred before [34].

The M1/M2 dichotomy of macrophages is a widely studied and applied concept [20]. M1 activation increases the expression of proinflammatory cytokines and production of ROS. In contrast, M2 may have anti-inflammatory and wound-healing properties. Exosomes from adipose-derived mesenchymal stem cells could ameliorate cardiac fibrosis after myocardial infarction by promoting M2 macrophage polarization [35]. In this study, we found aging increase the macrophages on aorta, but NAC did not affect it, but NAC did decreased the M1/M2 ratio. However, the simple characterization of M1 as bad and M2 as good is inaccurate. It is now clear that the M1/M2 dichotomy is not sufficient to describe the diverse phenotypes and functions of macrophages in vivo [36] where both M1 and M2 markers can be expressed simultaneously [37]. This phenomenon was also observed in our study; there was always a group of cells that expressed both CD80 and CD206 as determined by flow cytometry. At least, the M1/M2 ratio was higher in the older group and its correlation with collagen deposition suggests that inflammation is involved in the aging process and fibrosis development (Figure 3). M1 can switch to M2 [38], and high levels of M2 were expressed in NAC treatment group suggesting the anti-inflammatory effect of NAC on anti-fibrosis during the process of physiological aging and the possible mechanism underlying M2 polarization (Figures 3 and 4). However, some researchers indicated that continuous activation of M2 macrophages may impair tissue regeneration and function by promoting fibrosis [39,40]. Thus, further studies are required to compare the M2 function between the pathological and physiological processes, and to determine the role of NAC in macrophage polarization *in vitro*.

**Figure 5.**
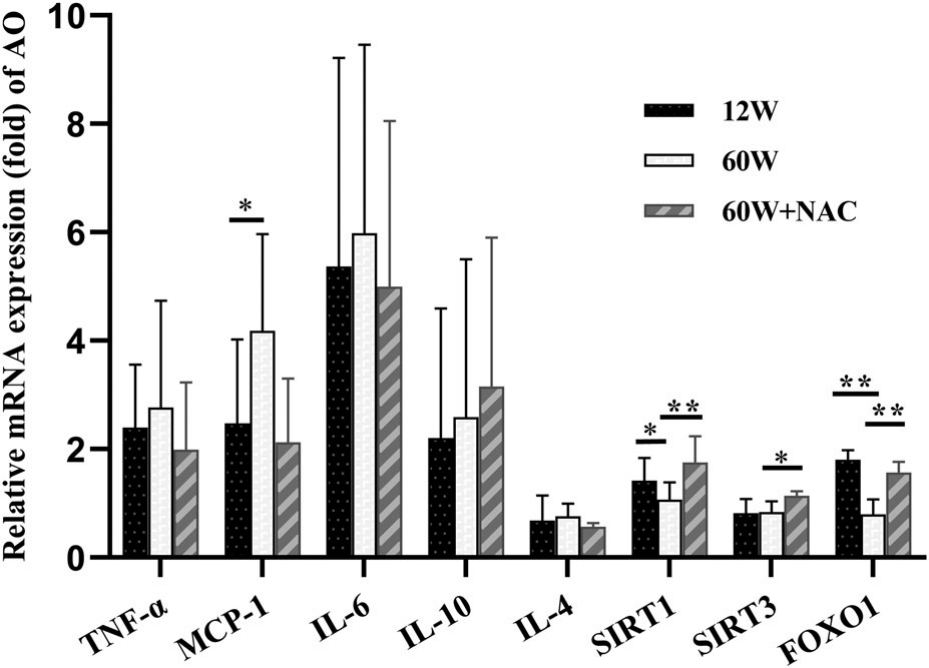
Genetic expression in aorta were detected by qRT-PCR. The aging group had a significantly higher MCP-1 mRNA level and lower SIRT1 and FOXO1 mRNA levels. The 12- week NAC treatment increased the genetic expressions of SIRT1, SIRT3, and FOXO1. Data were as means ± SD (*n* = 8 each). *p < 0.05, **p < 0.01.

Cytokines are key mediators that coordinate the processes of inflammation and repair in an injured muscle [29]. The expression of some proinflammatory cytokines such as TNF-α, IL-6, MCP-1, and IL-4, and the anti-inflammatory cytokine IL-10 in the aortic walls was analyzed in the present study (Figure 5). MCP-1 (CCL2) is one of the key chemokines that regulate migration and infiltration of monocytes/macrophages [41]. Only MCP-1 expression was significantly higher in the older group, partially because fibrosis is a chronic inflammatory process occurring during physiological aging and other cytokines are not as sensitive as MCP-1. Sirtuins are NAD^+^-dependent deacetylases, which modulate a wide range of biological processes, spanning from DNA repair and oxidative stress responses to energy metabolism. FOXOs belong to the family of forkhead proteins and are characterized by a highly conserved DNA binding domain (forkhead box). It controls various cellular responses, ranging from apoptosis, DNA repair, and metabolism to ROS detoxification and cell proliferation. The sirtuins and FOXO family members play important roles in various aspects of vessel growth, maintenance, and function [42,43]. Among them, some papers indicate that SIRT1, SIRT3, and FOXO1 may be related to fibrosis and inflammation in addition to vascular aging [44]. SIRT3 is related to cardiac hypertrophy and fibrosis [42], we detected the impact of NAC on above signaling, the expression of above signaling could all be upregulated post 12 week NAC treatment (Figure 5), hinting NAC may have other anti-inflammatory effects besides its impact on oxidative stress, such as promoting macrophage polarization. Both SIRT1 and SIRT3 could regulate FOXO activity. There was a strong and linear negative correlation between FOXO1 abundance and age in T cells and FOXO1 restricts endothelial angiogenic behavior in embryo and stem cells [42], which seems paradoxical as in aortas of our result. However, FOXO1 is needed for normal angiogenesis during wound healing through regulation VEGF and deletion of *Foxo1* in keratinocytes reduces re-epithelialization and granulation tissue formation [45]. That may explain why FOXO1 mRNA level expressed lower in aging aorta causing vascular fibrosis in this study and may be the possible mechanism that NAC could reverse it by increasing FOXO1 expression. Thus, further study is needed to clarify the detailed role of NAC and aging related fibrosis.

## Conclusion

In the physiological aging process in mice, 12-week NAC treatment ameliorated aortic fibrosis possibly by stimulating M2 macrophage polarization, and SIRT1, SIRT3 and FOXO1 may play a role in unidentified pathways linking vascular oxidative stress to fibrosis associated with aging.

## Supplementary Material

Supplemental Material
