# Supplementary material for: N-acetyl cysteine ameliorates aortic fibrosis by promoting M2 macrophage polarization in aging mice
Source: Redox Rep. 2021 Sep 17;26(1):170–5. doi: 10.1080/13510002.2021.1976568 (PMC8451627; doi:10.1080/13510002.2021.1976568)
Supplement: Supplemental Material [file YRER_A_1976568_SM3573.docx]

# Materials and Methods

# Animals and study design

Wild-type C57BL/6 mice were purchased from Hunan SJA Laboratory Animal CO (Changsha, P.R.China )and then bred for up to 24 months. They were maintained in a temperature-controlled facility with a strict 12-hour light-dark cycle and were provided food and water *ad libitum*. After sexual maturation by 21–24 days of age, the mice were segregated according to gender and each cage had five mice. Male mice were used in the subsequent experiments. After being weaned, the mice were fed a normal chow diet until 12 weeks (12 W) and 60 weeks of age. The latter were randomized into two groups when they were being fed until 48 weeks: They had free access to either deionized water (60 W) or water containing 1 mg/mL N-acetylcysteine (NAC, Sigma) (60 W+NAC) for 12 more weeks. This concentration of NAC has been shown to be effective in reducing the production of ROS in previous studies[1,2].Each group included 8 to 10 mice. The aortas were harvested and hematoxylin and eosin (H&E) staining and Masson’s trichrome staining was performed, and the levels of reactive oxygen species (ROS), RNA expression of GAPDH, TNF-α, MCP-1, IL-6, IL-10, IL-4, SIRT-1, SIRT-3, FOXO-1, and macrophage polarization were determined.

All animal experiments were performed in accordance with the “Guide for the Care and Use of Laboratory Animals of the Ministry of Health of China and the NIH guidelines of the care and use of laboratory animals.” The experimental protocols used in the present study were reviewed and approved by the Central South University Ethics Committee for Animal Research from The Second Xiangya Hospital (Changsha, P.R China).

# Tissue harvesting and aortic ring preparation

After isolating the mouse aorta, the adjacent adipose tissue was immediately removed with the help of a dissection microscope and kept in a Petri dish containing black dissection wax as described previously [3]. Sections of aortic rings on each slide were 6 mm long after the bifurcation of the subclavian artery. For macrophage staining, aorta cross-section was collected from near the aortic sinus. After freezing the aorta cross in 2-methylbutane in a beaker by placing it on dry ice, it was immediately embedded vertically in a labeled disposable freezing mold with optimal cutting temperature compound (OCT compound). When the OCT compound was totally frozen, the mold was transferred onto dry ice and then stored at −80°C by wrapping it with aluminum foil until sectioning was performed. After adjusting the chamber temperature to around −21°C, cryostat sections (7 μm) were prepared in 48 hours after isolation [4].

# H&E and Masson’s staining

After preparing the 7-μm cryostat sections, the aorta sections were stained with hematoxylin and eosin (#ab245880) or Masson’s trichrome stain (#ab150686) by following the manufacturer’s protocol. Collagen deposits were measured in randomly selected Masson’s trichrome-stained sections in 10 high-power (×400) fields. Photomicrographs were obtained using a digitalized microscopic image system ([Leica DM 5500B, Leica Microsystems,Wetzlar,](https://research.missouri.edu/mcc/leica_dm5500b_leica_dfc290cam.php) Germany). The images were analyzed using the Image J software[5]. For each study, analysis of histology sections was performed using at least 3 randomly chosen high-power fields from 3 different sections from each mouse.

# Immunofluorescence staining

# Immunofluorescence (IF) staining was performed according to “Cell Signaling Technology IF General Protocol”. Anti- alpha-SMA AF488 (#53-9760-80) at a concentration of 1:500 was purchased from Invitrogen™. F4/80-AF594 was used to represent total macrophages in our study. CD206-AF647 was used to represent M2 macrophages. Anti-F4/80 primary antibody (#ab100790) at a concentration of 1:100 was purchased from Abcam™ and its second antibody anti-rabbit AF594 (#A11072) at a concentration of 1:200 was purchased from Invitrogen™. DAPI (#D1306) and anti-CD206 AF647 (#141712) at a concentration of 1:200 were purchased from Invitrogen™. All the images were captured using the Leica TCP SP8 confocal microscope.

# ROS detection

The oxidative stress level in the cryostat sections of mouse aorta was assessed by confocal microscopy by using the dihydroethidium (DHE) dye as described previously [6]. The aortic segments and cryostat sections of mouse aortas were preincubated in DHE (5 μmol/L, Invitrogen, Carlsbad, CA, USA) 37°C for 15 min in physiological saline. At the end of incubation, the DHE dye was washed off and the fluorescence intensity of one optical section of the rings was visualized by using a laser scanning confocal system (Leica TCP SP8, Leica Microsystems). The fluorescence intensity was measured at 515 nm excitation and 585 nm emission wavelengths, and the images were analyzed using the Image J software.

# Flow cytometry for analyzing murine macrophages

To determine the number of macrophages in the aortic wall, the mice were sacrificed by CO_2_ asphyxiation at the endpoint and multicolor flow cytometric analysis was performed using an LSR Fortessa^™^ X-20 cell analyzer (BD Bioscience, CA, USA). The cells in the murine aorta were harvested by digestion as described previously [7]. Aortas were chopped into small pieces to improve enzymatic digestion (concentrated final digestion solution diluted in PBS: 450 U/mL collagenase type I, 125 U/mL collagenase type XI, 60 U/mL hyaluronidase type I-s, and 60 U/mL DNase-I of the tissues). The tissues were incubated for 45–60 min at 37°C and a 70-μm cell strainer and syringe were used to isolate the cells from the digested aortic tissue samples, after which the cells were transferred to an FACS tube and subject to flow cytometry. After gating the living cells with yellow LIVE/DEAD^™^ Fixable Dead Cell Stain Kits (Invitrogen) and gating leukocytes with CD45, the M1 population was identified by CD45^+^/CD107^+^/CD80^+^/ CD206^-^ and the M2 population was identified by CD45^+^/CD107^+^/CD80^-^/CD206^+^. Gates set up were based on FMO controls and debris was excluded based on the FSC/SSC profiles(not shown). All antibodies namely anti-CD45 APC-Cy7 (#103116) at a concentration of 1:100, anti-CD206 PE (#141706) at a concentration of 1:125, and anti-CD107b AF647 (#108512) at a concentration of 1:200 were obtained from Biolegend (San Diego, CA, USA) except for the Live and Dead kit BV605 that was obtained at a concentration of 1:1000 from Invitrogen (Carlsbad, CA, US) and anti-CD80 BV786 (#740888) obtained at a concentration of 1:125 from BD Pharmingen (San Jose, CA, USA). Flow cytometry data analysis was performed using the FlowJo software.

# Quantitative reverse transcription-polymerase chain reaction

Total RNA was isolated from the aorta of each mouse using Trizol (Sigma-Aldrich), followed by purification with RNeasy columns (Qiagen), and treatment with DNase I (Thermo Scientific). cDNA was synthesized by using 1 µg of total RNA in each sample by using the RevertAid RT Kit (Thermo Scientific). Intensity values were quantitated by using Applied Biosystems QuanStudio^™^ 3 Real-Time PCR System. The primers used are listed in Table 1.

# Table 1. Primers used for gene detection

| **Gene** | **Forward Primer** | **Reverse Primer** |
| --- | --- | --- |
| GAPDH | ACCCAGAAGACTGTGGATGG | CACATTGGGGGTAGGAACAC |
| TNF-α | CCCCTCTATTTATATTTGCACT | TATTTCTCTCAATGACCCGTA |
| MCP-1 | TACTCATTCACCAGCAAGAT | CAGATTTACGGGTCAACTTC |
| IL-6 | AGTTGCCTTCTTGGGACTGA | TCCACGATTTCCCAGAGAAC |
| IL-10 | TGCCTGCTCTTACTGACT | TCCACTGCCTTGCTCTTA |
| IL-4 | GCGACAAAAATCACTTGAGAG | CCTTGGAAGCCCTACAGAC |
| SIRT1 | ACCAGTAGCACTAATTCCAA | TATAACATCGCAGTCTCCAA |
| SIRT3 | CAGTATGACATCCCGTACCCT | GGCACTGATTTCTGTACTGCT |
| FOXO1 | GACAGTGGCAGGATTAGTTA | ATGGACGGAATGAGAGGTA |

**Statistics analysis**

SPSS 20.0 statistical software was used for performing data analysis. Measurement data were expressed as mean ± standard deviation (X±SD) using two-independent-sample t test (two-sided) for two groups of data or one-way analysis of variance (ANOVA) followed by post-hoc LSD test or Dunnett’s test for three or more groups. A *p-*value <0.05 was considered statistically significant.

**References**

1. Rocha-Vieira E, Ferreira E, Vianna P, et al. Histopathological outcome of Leishmania major-infected BALBef/c mice is improved by oral treatment with N-acetyl-l-cysteine. Immunology. 2003 Mar;108(3):401-8.

2. Sii-Felice K, Barroca V, Etienne O, et al. Role of Fanconi DNA repair pathway in neural stem cell homeostasis. Cell Cycle. 2008 Jul 1;7(13):1911-5.

3. Mohanta S, Yin C, Weber C, et al. Aorta Atherosclerosis Lesion Analysis in Hyperlipidemic Mice. Bio Protoc. 2016 Jun 5;6(11).

4. Kumar A, Accorsi A, Rhee Y, et al. Do's and don'ts in the preparation of muscle cryosections for histological analysis. J Vis Exp. 2015 May 15(99):e52793.

5. Schindelin J, Arganda-Carreras I, Frise E, et al. Fiji: an open-source platform for biological-image analysis. Nat Methods. 2012 Jun 28;9(7):676-82.

6. Lau YS, Tian XY, Mustafa MR, et al. Boldine improves endothelial function in diabetic db/db mice through inhibition of angiotensin II-mediated BMP4-oxidative stress cascade. Br J Pharmacol. 2013 Nov;170(6):1190-8.

7. Gjurich BN, Taghavie-Moghadam PL, Galkina EV. Flow Cytometric Analysis of Immune Cells Within Murine Aorta. Methods Mol Biol. 2015;1339:161-75.
